# The effect of educational intervention program on promoting preventive behaviors of urinary tract infection in girls: a randomized controlled trial

**DOI:** 10.1186/s12887-020-1981-x

**Published:** 2020-02-19

**Authors:** Zahra Ahmadi, Mohsen Shamsi, Nasrin Roozbahani, Rahmatollah Moradzadeh

**Affiliations:** 1 Department of Health Education, Faculty of Health, Arak University of Medical Sciences, Arak, Iran; 2 Department of Health Education and Health Promotion, Faculty of Health, Arak University of Medical Sciences, Arak, Iran; 3 Department of Epidemiology, Faculty of Health, Arak University of Medical Sciences, Arak, Iran

**Keywords:** Theory of planned behavior, Education, UTI, Randomized controlled trial

## Abstract

**Background:**

Urinary tract infection is one of the most common infectious diseases in children, which can lead to serious complications for a child. The purpose of this study was to investigate the impact of Theory Planned Behavior (TPB) -based education on the promotion of preventive behaviors of urinary tract infection in mothers with a daughter under age two.

**Methods:**

The present study is an educational randomized controlled trial that its sample consisted of 100 mothers who had a daughter under age two. They were selected through convenience sampling and then were randomly assigned to the intervention and control groups (each group included 50 participants). The data collection tool was a reliable and valid questionnaire based on TPB constructs. First, in both groups, the pre-test was administrated and then the educational intervention in the intervention group was conducted in the form of four educational sessions in 1 month (based on the pre-test need assessment) and then 3 months after the intervention (according to the ideas of Panel of Experts), post-test in both groups was administrated and then the data were analyzed through SPSS version 23 software with inferential statistics (independent t-test, paired t-test, and chi-square). The significance level was considered 0.05.

**Results:**

Three months after the intervention, the mean score of the constructs of TPB in the intervention group was significantly higher than the control group. The performance of prevention of urinary tract infection in the intervention group before the education increased from 2.85 ± 0.51 to 3.74 ± 0.29 (out of 4) (*p* = 0.001).

**Conclusions:**

TPB-based education with active and interventional follow-up was effective in promoting the preventive behaviors of urinary tract infection. Therefore, due to the side effects of UTI, especially in vulnerable periods such as childhood, it is recommended that trainings based on this model be carried out in other health care centers in order to maintain children health.

**Trial registration:**

This trial has been registered at IRCT, IRCT2017031533090N1. Registered on 9 July 2017, https://en.irct.ir/trial/25621

## Background

Urinary tract infection (UTI) is a term used for a wide range of clinical disorders, from asymptomatic bacteriuria to kidneys infection and sepsis. In more than 80% of cases, UTI is caused by a bacterium that the most commonly responsible organism for this bacterium is Ecoli which is part of the normal flora of the intestine [1]. The high prevalence of infection, the likelihood of recurrence of the disease, the variety of clinical symptoms in different age groups followed by the difficulty of clinical and laboratory diagnosis, the resistance of the causative agent to antibiotics and the long-term serious complications in children, have caused the infection of the urinary system to be of special importance in children [2, 3] so that nowadays, UTI is one of the most common bacterial infections in children and is considered as one of the most important health indicators of communities [4].

In the global reports reviewed in Uwaezuoke’s study, UTI prevalence rate range from as low as 6% to as high as 37% in developing countries [5] and in terms of gender in a meta-analysis study the prevalence of urinary tract infection was observed in 3% of girls and 1% of boys under age 10 [6, 7]. Brien also reported an UTI incidence of 5.9% and an estimated UTI incidence of 8% in children under age 5 in England [8].

The prevalence of this type of infection is different in different cities of Iran and is reported from 4 to 12%. The prevalence in girls is 77.2% more than boys and in terms of age, the highest prevalence is in the age group under 1 year old [9–11]. Out of 250 children with urinary tract infections aged 1 to 12 months who referred to Amir Kabir Hospital in Arak, 224 (89.6%) were girls and 26 (10.4%) were boys and the highest percentage was observed in girls [12].

In a study, urinary tract infection in female students was associated with factors such as the way of cleaning anus after fecal excretion, daily intake of water, previous history of dysuria, genital itching, frequent urination, and pain at the sides of the abdomen and in the lower abdomen [8, 11–13].

In India, in a study on urinary tract infection in 181 10–11 year-old adolescent girls in rural areas of Karimnagar, India, there was a significant relationship between the incidence of urinary tract infections and poor perineum cleaning, malnutrition, the presence of vaginal discharge, the use of unhealthy menstrual pads, and some misconceptions including not taking bath during menstruation period, which led to urinary tract infection [14].

Considering the high prevalence and serious complications of UTI in children, diagnosis and treatment of urinary tract infections as soon as possible with respect to health behaviors is essential. On the other hand, given the prominent role of adopting health behaviors in preventing urinary tract infections, educational interventions to improve the preventive behaviors of this infection are necessary [2].

The effectiveness of an educational intervention depends on an appropriate application of behavioral science theories. In this study, the Theory of Planned Behavior (TPB) has been used. TPB is one of the principal theories used to design the evidence-based interventions. This theory, in addition to paying attention to the attitudes of individuals, examines their behavioral intentions. Since most preventive behaviors of UTI in infants and children are carried out by the mothers at home and in privacy, investigating the behavioral intention and its improvement has a significant impact on the improvement of the proper behaviors of mothers in children care, especially in childhood and infancy [15].

According to this theory, the attitude of a person is a desirable or undesirable evaluation of a behavior that has been formed through mental perceptions or past experiences. Behavioral intention is the decision of an individual to adopt a behavior, and mental norms are the effects of different people on the behavior of an individual, and the extent to which the individual follows their opinions. Perceived behavioral control is the degree to which a person is capable of performing or not performing a certain behavior, in the control of his will [16].

Among similar studies based on TPB, a study conducted by Darabi et.al investigated the effect of health education program based on TPB on sexual and reproductive health in Iranian adolescent girls (12 to 16 years) and it showed the effectiveness of the educational program on the improvement of attitude, subjective norms, perceived behavioral control, behavioral intention, and behavior of adolescent girls [17]. Another study by Lee et al. on the application of TPB about predicting exercise intentions and behaviors of Taiwanese children indicated that perceived control behavior was the strongest predictor of the intention to exercise. This construct, affected the exercise behavior not only directly but also indirectly through intentions [18].

Moreover the study of Duncanson et al. on parents’ perceptions of child feeding- a qualitative study based on TPB- indicated that the application of TPB to child feeding may explain the disparity between parents’ child-feeding intentions and behaviors. Parents’ feeding behaviors are more influenced by peers than by dietary guidelines [19].

Considering that children are the most sensitive group to infectious diseases and that the previous studies on this problem (2,3,8,9,10,14, 20) that investigated the effective factors on urinary tract infection were review, survey, qualitative, prevalence measurement, and descriptive studies. This study (educational randomized controlled trial) has been conducted based on TPB and emphasizes on constructs “attitude”, “perceived behavior control”, “subjective norm” and “intention behavior” beside a 3 month follow-up for durability of mothers’ behavior to prevent urinary tract infection in their daughters and this is the novelty of this study.

Since in the previous studies, no study has been conducted on the use of TPB in improving the preventive behaviors of UTI in mothers (as the most important and the first child health care provider) who have a daughter under age two, the present study aimed at investigating the effect of education based on TPB on the adoption of preventive behaviors of urinary tract infections in mothers who have a daughter under age two in order to promote children’s health.

## Methods

### Study design

**Fig. 1:**
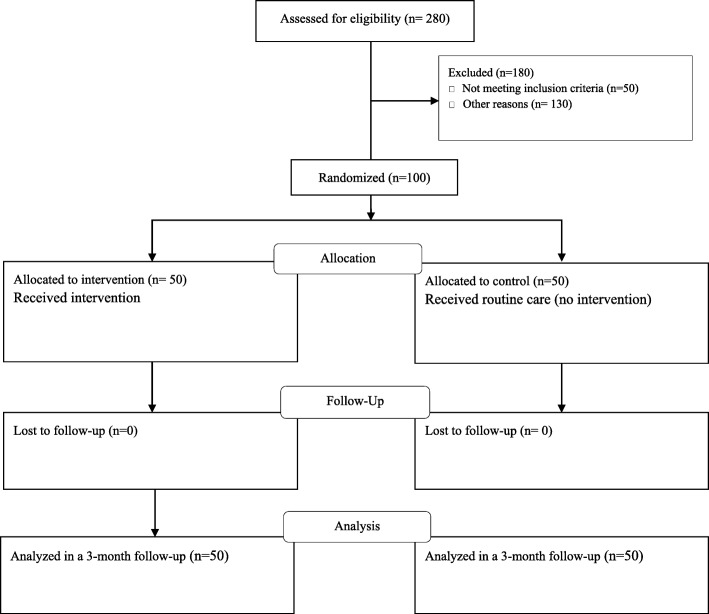
Flow of participants. From a total of 280 mothers with daughters under age two, 100 who met the inclusion criteria were randomly assigned into intervention and control groups (each group 50). Pre-test was administrated in both groups and then the intervention group received TPB-based training and the control group received routine training of health centers. Both groups were followed up for 3 months and finally in both groups post-test was administrated and the data was analyzed. The results showed that the improvement of urinary tract infection preventive behaviors in the intervention group was significantly higher than the control group

The present study is an educational randomized controlled trial. From a total of 280 mothers with daughters under age two, 100 who met the inclusion criteria were randomly assigned into intervention and control groups (each group 50). Then the pre-test was administered to both groups based on the questionnaire. The intervention group received trainings based on TPB and the control group received routine cares. Then the mothers were followed up for 3 months. After that the post-test was administrated and finally the effect of education on their preventive behaviors was re-evaluated. Figure 1 shows the flow diagram of the participants during the study.

The criteria for entering the study were mothers who were literate (with minimal reading and writing ability) and their daughter was healthy (or if they had UTI, their numbers in both intervention and control groups were the same).

The exclusion criteria of the study included lack of continuous presence of the mothers in educational intervention, reluctance to continue participation in the study, and lack of availability of mothers when completing the post-test questionnaire.

### Sample sizing estimation

According to the study [20] and the following formula and with regard to the mean and standard deviation of “behavior” that was 8 ± 2 before the intervention and 9 ± 1. 42 after the intervention, and alpha = 0.05 and β = 0.2, the sample size was 47 in each group. Considering the dropout rate, finally 50 people in each group entered the study.

$$ n=\frac{\left({s}_1^2+{s_2}^2\right){\left({z}_{1-\frac{a}{2}}+{z}_{1-\beta}\right)}^2}{{\left(\overline{x_1}-\overline{x_2}\right)}^2} $$

To sample, the city first was divided into four areas according to the geographical map and then based on the list of health centers in that area, two health centers were randomly selected from each region and in total eight health centers were selected in the whole city. Half of the selected health centers in each region were randomly assigned to the intervention group and the other half to the control group, and the samples, available in each center, were included in the study. The intervention group was influenced by a planned educational program based on TPB and the control group was influenced by the routine education of the health centers.

According to the opinions of Panel of Experts, 3 months of follow-up was considered sufficient time to establish consistency, stability, and sustainability in promoting preventive behaviors of urinary tract infection in girls.

Therefore 3 months later, the impact of the educational program on prevention of urinary tract infection in children were evaluated and compared in both groups. Based on the nature of the intervention in the present study, the instructor was not blinded to group assignment, but participants and statistical investigator were blinded to group assignment.

Three months after the intervention, the post-test was administered in both experimental and control groups to examine the effects of education on the primary and secondary outcomes.

The primary outcomes of the current research included the constructs of TPB (Attitude, Subjective norm, Perceived Control Behavior, Intention) and the secondary outcome was urinary tract infection prevention behavior in children.

## Measures

The data collection instrument was a reliable and valid questionnaire including the following sections:

1. Demographic questionnaire: including mother’s age, occupation, education, number of family members, number of children, child care, the history of urinary tract infection in her child, and her child’s age, weight, and nutrition status.
2. Questionnaire for knowledge: Mothers’ Knowledge on prevention of UTI in children: Including 12 Multiple-choice questions. For example, “which of the symptoms of urinary tract infection are seen in infants and children under age two?”
3. Theory of Planned Behavior questionnaire: The constructs of Theory of Planned Behavioral include:
  A. An instrument for measuring the attitude of mothers about preventing urinary tract infections in children: Includes 14 questions with 5-point Likert spectrum (totally agree, agree, no opinion, disagree, totally disagree). For example, “Urinary tract infection is a serious and dangerous disease.”
  B. An instrument for measuring mothers’ perceived behavioral control in prevention of children urinary tract infection: six questions were designed with 5-point Likert spectrum. Perceived behavioral control of the belief in mothers depends on the ease or difficulty of performing the preventive behaviors of urinary tract infection under every circumstance. For example, “I can shower her without putting my baby in the pelvis or tub.”
  C. An instrument for measuring mothers’ subjective norms in prevention of UTI: For this purpose, nine questions with 5-point Likert spectrum were designed. For example, “My husband encourages me to use urinary tract infection behaviors in my child.” Subjective norm is the extent to which a mother is influenced by others to take UTI preventive behaviors in her child.
  D. A measuring instrument for mothers’ intention for prevention of urinary tract infection: includes five questions with 5-point Likert spectrum. For example, “I’m going to do the right front-to-back cleaning way of anus for my child.” Behavioral intention in this study refers to the mothers’ attitudes toward prevention of urinary tract infection in daughters under age two.
  E. A measuring instrument for mothers’ behavior in preventing urinary tract infection in children: Includes fourteen questions with 5-point Likert spectrum (always, often, sometimes, rarely, never), for example “using a sanitary toilet paper to dry your child’s urethral area after urinating or conducting some behaviors like feeding baby with breast milk instead of baby formula”. In this study, preventive behaviors for urinary tract infections include changing baby diapers pretty soon after bowel movements, washing hands before cleaning the baby’s urethral area, using sanitary toilet papers, and visiting a doctor if a mother sees the symptoms of urinary tract infection in the child, all of which were self-reported.

According to Ajzen, using the self-report method and relying on individual’s reports rather than direct observation and immediate measurement of the target behavior, due to some problems in obtaining data in the time limits, is an accepted method in TPB studies [21, 22].

To score the questionnaire in the knowledge section, for correct answer the score was 1, and for wrong answer the score was zero and the scores ranged from a minimum of zero to a maximum of one. In the performance evaluation section for doing each behavior, the average scores of the responses based on a 5-scale spectrum was calculated as follows: Never = 0, rarely = 1, sometimes = 2, often = 3, always = 4 and the scores ranged from a minimum of zero to a maximum of four. Questions of attitude, perceived behavior control, and behavioral intention were scored using 5-point Likert Scale as follows: Strongly disagree = 1, disagree = 2, no opinion = 3, agree = 4, strongly agree = 5 and the scores ranged from a minimum of one to a maximum of five.

### Validity and reliability of the questionnaire

Validity of the questionnaire was assessed through content validity in two quantitative and qualitative ways and with the assistance of ten professors and specialists in health education and promotion, midwifery, kidney and urology, and pediatrics. In the quantitative assessment of content validity, the Content Validity Ratio (CVR) and Content Validity Index (CVI) were calculated. The criterion for accepting the items in the Content Validity Index was approved with an index of at least 0.79 and in the Content Validity Ratio, according to the Lawesh’s Table Scale and the number of Specialists, was approved with a reliability of at least 0.62 [23].

Finally, content validity rate and content validity index were 0.83 and 0.91, respectively.

To determine the reliability of the questionnaire, the internal consistency method was used on 30 mothers who were similar to the studied population in terms of the demographic characteristics. Finally, the internal consistency obtained from Chronbach’s alpha revealed that all of the coefficients were desirable and satisfactory so that the knowledge coefficient was 0.79, the attitude coefficient was 0.85, the subjective norm coefficient was 0.76, the perceived behavior control coefficient was 0.81, the behavior intention coefficient was 0.92, and the performance assessment coefficient was 0.71.

### Educational intervention

At first, the pre-test was administrated in both groups and based on this needs assessment, the theory-based education program was designed and developed. In the educational intervention a variety of communication techniques was used to encourage mothers to participate in the intervention. The results of the need assessment (pre-test) provided helpful information to design and develop the intervention program.

Various educational strategies were used to determine the behavioral goals in each of the learning areas and constructs of TPB including: knowledge section (lectures, question and answer sessions, and slideshows), attitude section (group discussion, role-playing, brainstorming, and verbal encouragement), and subjective norms section that refers to the beliefs of mothers about whether significant others (spouses, health workers, physicians, and so on) think she will perform the urinary tract infection behaviors in children (by providing information, guidance materials in booklets and pamphlets for the spouses), and perceived behavioral control section which increases when mothers perceive they have more resources and self-confidence. In the intervention program, we tried to increase mothers’ perceived behavior control by providing information through lectures, small group discussion, and verbal encouragement.

Moreover a booklet was developed and adapted for mothers’ reading ability level. The final draft of the booklet was evaluated by mothers outside the present study before applying to the intervention. The information in the booklet was based on TPB model constructs and was written in a simple and understandable language.

The educational intervention was carried out in the form of 4 educational sessions for 1 month and as follows:

In order to promote the knowledge of mothers, the educations were about the introduction of the structure of the kidney and urinary tract, the description of urinary tract infection, informing about the symptoms of urinary tract infection in children, risk factors for urinary tract infection, the prevention of urinary tract infections in children and so on.

The educational plan was implemented using direct and indirect (booklets, pamphlets, slide shows) methods for the intervention group. Direct methods were applied to enhance the knowledge of mothers about urinary tract infection, side effects of UTI (the possibility of children being more vulnerable and at risk), and to correct mothers’ mistakes through lectures and question and answer sessions, etc.

In one of the educational sessions that was designed to improve the attitude of mothers towards preventing urinary tract infection in children, in order to influence this construct, according to the process recommended by Sharma et al. [24], the method of brain storming and group discussion was used to correct or change the wrong beliefs and to strengthen the right beliefs and it was emphasized that mothers should believe that urinary tract infection is a child’s health threat and that urinary tract infection in children can have serious consequences for them.

In another educational session, in order to change the subjective norms, we used role play and group discussion which led the mothers to know what others think about them. We tried to emphasize the views of specialists, doctors, and health care providers about preventive behaviors. Also a pamphlet entitled as “UTI in Children” was given to the mothers and their husbands. By doing this, mothers’ families had detailed information about the urinary tract infection, and they would be more likely to confirm their behaviors, and then the mothers would be more likely to do performable and manageable behaviors.

For perceived behavioral control, different steps were taken including: discussing about the facilitators of the behavior, providing incentives, reducing and eliminating the barriers, breaking the behavior into small steps, using educational photos and videos, and using experiences of the mothers who have had proper behaviors to prevent urinary tract infection.

In order to promote the behavior of mothers, the followings were taught to the participating mothers: the necessity for timely urinalysis at age one, the importance of prescribing the medication only with the advice of a physician to prevent and control urinary tract infections, emphasizing the ease of prevention behaviors, the impact of health behaviors on reducing the incidence of urinary tract infections in children, and the correct way of doing the right behaviors (using power point presentations).

Regarding the nature of preventive behaviors of urinary tract infection in children and based on the opinions of Panel of Experts, 3 months of follow-up was considered sufficient time to establish consistency, stability, and sustainability in promoting preventive behaviors of Urinary Tract Infection in girls. Finally, 3 months after the intervention, the post-test was administrated and the data were collected from both groups and were analyzed.

### Statistical analysis

Data analyses were performed using IBM SPSS Statistics for Windows, Version 23 (Armonk, NY: IBM Corp) through descriptive and inferential statistics (including independent t-test, paired t-test, Chi-square). The significance level was considered at 0.05. To investigate the normality of the data, Kolmogorov-Smirnov test was used and normal distribution of the data was obtained.

### Ethical considerations

Written informed consent was obtained from all the participants. Moreover, after the study, the training materials such as the booklets were given to the control group.

The present study was approved by ethics committee of Arak University of Medical Sciences (code: IR.ARAKMU.REC.1395.377) and registered in Iran Registry Clinical Trials (code: IRCT2017031533090N1).

## Results

### Sample characteristics

The average age of the mothers was 30.4 ± 5.1 years in the intervention group and 31.1 ± 4.6 years in the control group (*p* = 0.501) and the average age of the children was 10.4 ± 4.7 months in the intervention group and 13.6 ± 6.5 months in the control group (*p* = 0.07), which did not have a significant difference. Also, the average weight of the children in the intervention and control groups was 8.5 ± 2.3 and 8. 9 ± 2.4 kg, respectively (*p* = 0.397). Other specifications of the units under study have been presented in Table 1.

**Table 1:** Comparison of the intervention and control groups, concerning the demographic variables

Variable	Group	Control		Intervention		*P*- Value
		Frequency (*N*)	Percent (%)	Frequency (*N*)	Percent (%)	
Education level of mothers	Elementary	4	8	3	6	0.71
	High school graduate	20	40	24	48	
	College education	26	52	23	46	
Education level of husbands	Illiterate	0	0	2	4	0.18
	Elementary	2	4	5	10	
	High school graduate	22	44	25	50	
	College education	26	52	18	36	
Number of children	two or less	21	42	24	48	0.61
	More than two	29	58	26	52	
Occupation of mother	Employed	4	8	3	6	0.5
	Housewife	46	92	47	94	
A history of UTI in child	Yes	7	14	12	24	0.22
	No	39	78	31	62	
	I don’t know	4	8	7	14	
Birth order	First	21	42	26	52	0.48
	Second	27	54	21	42	
	Third or more	2	4	3	6	

### Evaluation of intervention

The difference between two groups has been shown in Table 2. After the educational intervention, the paired t-test showed a significant difference between the mean score of the variables in the intervention group before and after the intervention, while there was no significant difference in the control group. Independent T-test showed that the mean score of attitude, subjective norm, behavioral control, and behavioral intention and mothers’ performance in relation with the preventive behaviors of urinary tract infections in children before the intervention in the intervention and control groups did not have a significant difference, but 3 months after the educational intervention, the difference was statistically significant. The performance of the prevention of urinary tract infection in the intervention group before the education increased from 2.85 ± 0.51 to 3.74 ± 0.29 (out of 5) (*p* < 0.001) (Table 2).

**Table 2:** Comparison of the intervention and control groups, concerning the TPB, before and after the intervention

Variable	Group	Intervetion		Control		*P*-value^a^
		Mean	SD	Mean	SD	
Knowledge	**Before**	0.62	0.16	0.58	0.15	0.2
	**After**	0.97	0.06	0.52	0.15	0.001
	**P-value**^**b**^	0.001		0.08		
Attitude	**Before**	3.89	0.36	3.8	0.33	1
	**After**	4.60	0.28	3.86	0.40	0.001
	**P-value**^**b**^	0.001		0.53		
Subjective norms	**Before**	4.15	0.43	4.10	0.49	0.63
	**After**	4.46	0.38	4.01	0.44	0.001
	**P-value**^**b**^	0.001		0.20		
Perceived behavior control	**Before**	4.41	0.47	4.37	0.45	0.61
	**After**	4.70	0.34	4.35	0.4	0.001
	**P-value**^**b**^	0.001		0.73		
Behavior intention	**Before**	4.63	0.43	4.61	0.42	0.82
	**P-value**^**b**^	4.86	0.28	4.56	0.46	0.001
	**After**	0.001		0.41		
Performance	**Before**	2.89	0.54	2.85	0.51	0.7
	**After**	3.74	0.29	2.8	0.48	0.001
	**P-value**^**b**^	0.001		0.96		

^a^Independent t test

^b^Paired t test

## Discussion

The aim of this study was to investigate the impact of Theory Planned Behavior based education on the promotion of preventive behaviors of urinary tract infection in mothers who have a daughter under age two.

In the present study, the educational intervention based on TPB could improve the performance of mothers in prevention of urinary tract infection in the intervention group compared to the control group.

Improvement of knowledge in the intervention group compared to the control group after the intervention was due to the educating mothers about preventative behaviors of UTI. Meanwhile, there were some tips and materials in the booklet and the educational pamphlet for them, and these factors improved the knowledge of the mothers in the intervention group. These findings are consistent with the interventional studies on the other health fields [20, 25, 26].

In the study of Jalali et al., lack of knowledge and non-observance of some health advice about sexual behaviors were related to urinary tract infection [16].

In this study, before the intervention, the mean score of attitude was 3.9, which increased to 4.6 after the intervention. However, the mean score of attitude in the control group did not change. This finding is consistent with the results of some studies such as Naseri-Salahshour [27], Darabi et al. [17]. This increase can be attributed to the promotion of the mothers’ attitudes about seriousness of UTI and paying attention to its complications and the cost of treatment. In fact, researchers believe that having knowledge alone is not enough to take preventive behaviors. But an individual’s attitude towards a disease is an important factor in conducting preventive behaviors. Also, in a study by Zhang et al., the reduction of negative attitudes of mothers in infant feeding leads to the improvement of their performance in caring behaviors (feeding) for their children [28]. In a study based on TPB in Netherlands, attitude significantly predicted individual health behaviors [29].

In the studies mentioned, there was a positive relationship between attitude and performing health behaviors, which indicates the importance of this factor. In the present study, using group discussions and experiences of mothers and motivating them to prevent the occurrence of urinary tract infections in children and introducing the benefits of these behaviors and the complications due to the lack of this care, ultimately increased the attitude of mothers after the intervention.

Another finding of this study was the enhancement of subjective norm construct in the intervention group after implementation of the educational program. This finding was consistent with a study by Jalali et al., on promoting the preventive behaviors of urinary tract infection in pregnant women [16] and with another study by Darabi et al. [17] on the promotion of preventive behaviors in the adolescence girls. Also, in another study on urinary tract infections in pregnant women, 71.8% of the pregnant women under study accepted family advice about prevention of UTI [30].

In the present study, physicians, husbands, health personnel, and families were influential in increasing the motivation of mothers to follow these individuals. Hence guidance from the family, doctors, or health care providers based on the theory of planned behavior in form of educational classes during the early years of childhood life, particularly to prevent urinary tract infection in girls under age two, will be very helpful. Therefore in this study, in order to increase the information of the influential people, some of the materials of the educational booklet were specifically designed on increasing the information of households and especially the husbands to be effective in adopting the behaviors by mothers.

The results of this study showed a significant difference in the perceived behavioral control scores of the experimental group before and after the intervention. In the study of Soltani et al., the importance of this construct in changing the behavioral intention of children has been emphasized [31]. In Jalali’s study [16], the perceived behavioral control for prevention of urinary tract infection increased after the intervention. In this respect, as the studied groups are different, they are not comparable with regard to the nature of the subjects and participants under study.

In the theory of planned behavior, higher perceived behavioral control increases the positive feeling toward the desired behavior and reduces the perceived barriers. If this happens along with the facilitators of the behavior, and controlling behavior is more consistent with actual control, the intention will be higher and the likelihood of the behavior will increase. Therefore, perceived behavioral control has direct and indirect effects on the target behavior [32] As Ajzen [22] suggests that in the theory of planned behavior, the control of actual behavior is the extent to which a person has the skills, resources, and other prerequisites for a certain behavior. Therefore, the successful performance of a certain behavior does not depend only on having a favorable level of a desirable intention for the individual but also depends on the acceptable level of behavioral control.

In the present study, in order to increase perceived behavioral control, we tried to resolve some of the attitude barriers in the minds of the mothers in the educational intervention by introducing group discussions and using the experiences of other experienced mothers. In this regard, we tried to inform the mothers about the lack of knowledge about their care and to increase their skills and abilities to prevent urinary tract infection in their children, and we also addressed mothers’ ambiguities in this regard.

The results of the study showed that the educational intervention was effective in changing the behaviors of the mothers after the intervention compared to before the intervention (the mean was 2.89 before the intervention and it increased to 3.74 after the intervention). In fact, this improvement of behavior can be related to new educational methods and using health education theories and models. This finding is consistent with the results of some interventional studies such as Zhang et al. [28], Karimi et al. [33] that are conducted based on the model.

In a study conducted on the impact of the pattern of planned behavior on promoting the preventive behaviors for urinary tract infections in pregnant women who went to health centers, similar results were obtained regarding the effectiveness of the educational intervention in the control and intervention groups on the promotion of the prevention of urinary tract infection in pregnant women [16]. It is worth noting that the study groups were different from the present study. However, the promotion of prevention behaviors in both studies is due to the educational interventions.

In this study, the promotion of preventive behavior of mothers due to the educational intervention in improving mothers’ behavior included teaching about changing baby diapers pretty soon after bowel movements, how to put a baby in the bathtub correctly while bathing, the correct way of bathing a baby using the shower, washing hands before bathing a baby, breastfeeding, etc., which were taught to the mothers using slideshows and pictures and the mothers’ questions were also answered.

### Strengths and limitations

One of the strengths of the present study is that the design of the educational intervention for the prevention of urinary tract infection in children was based on a the need assessment (pre-test) and the constructs of the theory of planned behavior, as well as on following up of the behavior of mothers 3 months after the educational intervention.

The present study had some limitations. The most obvious limitation of the present study is the collection of information using self-report. This limitation was resolved by allocating sufficient time and explicit expression of the objectives of study, and gathering information along with interviewing. Also, we followed up the mothers for 3 months as the longer follow up may lead to more accurate outcomes.

This study showed that educational intervention based on TPB was effective in changing the behaviors of the mothers. However, whether mothers’ behavioral changes directly have clinical implications for the prevention of urinary tract infection in their children requires further studies. In other words, more evidence is needed to show that TPB can actually be effective in preventing urinary tract infections in this population. It is suggested that future studies be designed to use UTI laboratory tests in addition to questionnaires to assess the impact of health behaviors.

## Conclusion

According to the results of the education based on TPB, when mothers become aware of children urinary tract infection and feel their children are at risk of developing the disease and take the risk of it seriously, then they will have a high understanding of preventative behavioral control of urinary tract infection, which will make them feel less of a barrier to doing preventive behavior and therefore conducting preventive behaviors for urinary tract infections by them will increase. It is suggested that the efficiency and effectiveness of interventions based on Theory of Planned Behavior in the prevention of urinary tract infections in other settings (clinics, offices, etc.), as well as the evaluation of long-term effects (more than 1 year) in future studies be examined.

## Abbreviations

CVI: Content Validity Index
CVR: Content Validity Ratio
TPB: Theory of Planned Behavior
UTI: Urinary Tract Infection
